# 1,3‐Bis(aryloxy)propan‐2‐ols as potential antileishmanial agents

**DOI:** 10.1111/cbdd.13024

**Published:** 2017-06-28

**Authors:** Stefânia N. Lavorato, Mariana C. Duarte, Daniela P. Lage, Carlos A. P. Tavares, Eduardo A. F. Coelho, Ricardo J. Alves

**Affiliations:** ^1^ Departamento de Produtos Farmacêuticos Faculdade de Farmácia Universidade Federal de Minas Gerais Belo Horizonte Brazil; ^2^ Departamento de Patologia Clínica COLTEC Universidade Federal de Minas Gerais Belo Horizonte Brazil; ^3^ Programa de Pós‐Graduação em Ciências da Saúde: Infectologia e Medicina Tropical Faculdade de Medicina Universidade Federal de Minas Gerais Belo Horizonte Brazil; ^4^ Departamento de Bioquímica e Imunologia Instituto de Ciências Biológicas Universidade Federal de Minas Gerais Belo Horizonte Brazil

**Keywords:** antileishmanial activity, bisaryloxyalcohols, cytotoxicity, *Leishmania amazonensis*, leishmaniasis

## Abstract

We describe herein the synthesis and antileishmanial activity of 1,3‐bis(aryloxy)propan‐2‐ols. Five compounds (**2**,** 3**,** 13**,** 17**, and **18**) exhibited an effective antileishmanial activity against stationary promastigote forms of *Leishmania amazonensis* (IC_50_ < 15.0 μm), and an influence of compound lipophilicity on activity was suggested. Most of the compounds were poorly selective, as they showed toxicity toward murine macrophages, except **17** and **18**, which presented good selective indexes (SI ≥ 10.0). The five more active compounds (**2**,** 3**,** 13**,** 17**, and **18**) were selected for the treatment of infected macrophages, and all of them were able to reduce the number of internalized parasites by more than 80%, as well as the number of infected macrophages by more than 70% in at least one of the tested concentrations. Altogether, these results demonstrate the potential of these compounds as new hits of antileishmanial agents and open future possibilities for them to be tested in *in vivo* studies.

## 1 INTRODUCTION

Leishmaniasis is a neglected tropical disease caused by more than twenty species of *Leishmania* protozoa and is transmitted to mammals by the bite of the infected phlebotomine sandflies. This disease is responsible for 20,000–30,000 deaths annually and it is estimated that 310 million people live in areas at risk of infection, spread through 98 countries in the world.^1^

The treatment of leishmaniasis is restricted to a few drugs, such as pentavalent antimonials, amphotericin B, pentamidine, paromomycin, and miltefosine, used alone or in association. All these drugs are not ideal for the treatment of the disease due to their high toxicity and costs, long treatments, and the emergence of parasites resistance cases.^2^, ^3^ Therefore, the search for new potent and safe therapeutic alternatives is urgently needed.

1,3‐Bis(aryloxy)propan‐2‐ols are compounds easily obtained by synthesis; hence our interest is in exploring their pharmacological potential. A survey of the literature on their biological uses revealed that some compounds with this structural pattern have been exploited as antimycobacterial^4^ and antiasthmatic agents^5^ and as trimethoprim‐resistant dihydrofolate reductase inhibitor.^6^ However, to the best of our knowledge, their antileishmanial activity has not been evaluated so far.

In this context, in the present work, a series of 1,3‐bis(aryloxy)propan‐2‐ols was prepared and submitted to the evaluation of their antileishmanial potential. The *in vitro* antileishmanial activity was assessed against stationary‐phase promastigotes of *Leishmania amazonensis*, and the cytotoxicity was evaluated in murine macrophages. In addition, the compounds displaying better antileishmanial activity were selected to the investigation of their antileishmanial effect on intramacrophage amastigotes of the parasites.

## 2 EXPERIMENTAL

### 2.1 General

Reactants were obtained from commercial suppliers and used without further purification. Melting points were determined on Microquímica MQAPF 301 apparatus. The ^1^H and ^13^C NMR spectra were obtained on a Bruker Avance DPX‐200 spectrometer. The proton and carbon chemical shifts (δ) are given with respect to TMS. IR spectra were recorded on a Spectrum One, PerkinElmer ATR system. Column chromatography was performed on silica gel 60 0.063–0.200 mm/70‐230 mesh Merck. Anhydrous DMSO was dried over 3 Å molecular sieves.

### 2.2 Synthesis

Procedures for synthesis of compounds **1**–**21** and characterization data are shown in Supporting information.

### 2.3 ClogP determination

The calculated partition coefficient (ClogP) of each compound was determined using ACD/ChemSketch software (acdlabs.com).

### 2.4 Parasites and mice

*Leishmania amazonensis* (IFLA/BR/1967/PH‐8) strain was used in this study. Parasites were grown at 24°C in Schneider's medium (Sigma, St. Louis, MO, USA), supplemented with 20% heat‐inactivated fetal bovine serum (FBS, Sigma), 20 mm l‐glutamine, 200 U/ml penicillin, and 100 μg/ml streptomycin, pH 7.4. Stationary‐phase promastigotes were prepared as described previously.^7^ Murine peritoneal macrophages were obtained from female BALB/c mice, which were purchased from the Institute of Biological Sciences from UFMG. The experimental protocol was approved by The Animal Use Committee from UFMG (CETEA/UFMG) (code 136/2012).

### 2.5 In vitro antileishmanial activity

The concentration needed to inhibit 50% of *Leishmania* viability (IC_50_) was assessed *in vitro* by cultivating stationary promastigotes (1 × 10^6^ cells) in the presence of a serial dilution of each individual compound (50.0–0.1 μg/ml) in 96‐well culture plates (Nunc, Nunclon^®^, Roskilde, Denmark), for 48 hr at 24°C. Amphotericin B (AmpB) (10.0–0.1 μg/ml) was used as a control. Cell viability was assessed by measuring the mitochondrial oxidative activity with MTT [3‐(4.5‐dimethylthiazol‐2‐yl)‐2.5‐diphenyl tetrazolium bromide]. Absorbances were measured using a multiwell scanning spectrophotometer (Molecular Devices, Spectra Max Plus, Canada), at a wavelength of 570 nm. The compounds IC_50_ values were determined by applying the sigmoidal regression of concentration–response curves, using the different tested concentrations. Data shown are representative of three independent experiments, performed in triplicate, which presented similar results.

### 2.6 Cytotoxicity evaluation and selectivity index

Peritoneal macrophages were obtained from female BALB/c mice, which received an injection with 3% sodium thioglycolate and 4 days after the peritoneal cavity was washed with 8 ml sterile RPMI 1640 medium. Then, the cell suspension was adjusted to 1 × 10^6^ macrophages per ml. The concentration needed to inhibit 50% of macrophages viability (CC_50_) was evaluated by incubating these cells (5 × 10^5^) with a serial dilution of each individual compound (50.0–0.1 μg/ml) in 96‐well plates, for 48 hr at 37°C. AmpB (10.0 μg/ml) was used as a control. Cell viability was assessed by measuring the mitochondrial oxidative activity with MTT, and absorbance was measured using a multiwell scanning spectrophotometer, at a wavelength of 570 nm. In addition, the selectivity index (SI) was calculated by the ratio between the CC_50_ and IC_50_ values.

### 2.7 Treatment of infected macrophages

Macrophages (5 × 10^5^ cells) were plated on round glass coverslips inside the wells of 24‐well culture plates (Nunc) with RPMI 1640 medium, which was supplemented with 20% FBS and 20 mm l‐glutamine, pH 7.4. After 2 hr of incubation at 37°C in 5% CO_2_, stationary‐phase promastigotes of *L. amazonensis* (5 × 10^6^ parasites) were added to the wells and the cultures were incubated for 24 hr at 37°C in 5% CO_2_. Next, free parasites were removed by extensive washing with RPMI 1640 medium and treated with selected compounds (15.0, 10.0, 5.0 and 2.5 μg/ml) or AmpB (10.0 μg/ml), for 48 hr at 37°C in 5% CO_2_. After, cells were washed, set, and stained to determine the percentage of infected macrophages by counting 300 cells in triplicate. Data shown are representative of four independent experiments, performed in triplicate, which presented similar results.

## 3 RESULTS AND DISCUSSION

### 3.1 Chemistry

For the twenty‐one 1,3‐bis(aryloxy)propan‐2‐ol derivatives, different substituted aromatic rings were selected, which would provide us with compounds possessing a variety of physicochemical features such as electronic distribution, hydrophobicity and molecular volume, to establish a structure–activity relationship to shed some light on the importance of aryloxy group to their biological activity.

**Scheme 1:**
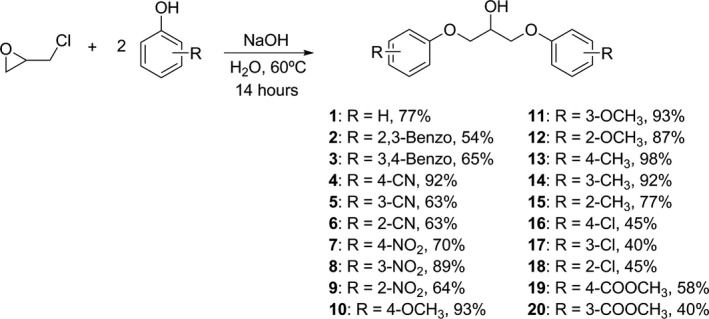
Synthesis of 1,3‐bis(aryloxy)propan‐2‐ols **1**–**20** and yields

Except for **21**, the proposed compounds were obtained by the reaction of epichlorohydrin with the appropriate phenol in alkaline medium,^8^ as shown in Scheme 1. The reaction of equimolar amount of epichlorohydrin and phenols provides the phenylglycidyl ether, which is an important synthetic intermediate of several drugs, like beta‐blockers propranolol^9^ and atenolol.^10^ As the purpose was to obtain symmetrical bis‐substituted compounds, the molar relationship between these two reagents was adjusted accordingly and the proposed compounds were obtained in a single step.

**Scheme 2:**
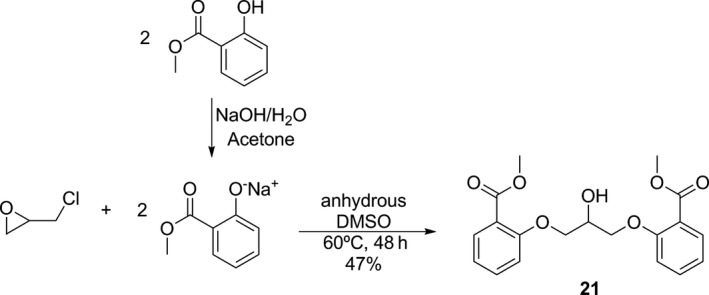
Alternative synthesis to obtain compound **21**

The best yields were achieved with a reaction time of 14 hr and they varied from 40 to 98%. Compound **21** (R = 2‐COOCH_3_) could not be obtained by the general method. A reason to explain this fact may be the formation of a chelate between sodium and methyl salicylate,^11^ which contributes to reduce the phenol reactivity and, consequently, the product formation. Applying some modifications in the general method based on these inferences, **21** was obtained in 47% yield by reacting epichlorohydrin with previous prepared sodium 2‐methoxycarbonylphenolate in anhydrous DMSO (Scheme 2).

### 3.2 Biological studies

After characterization, synthesized compounds had their antiparasitic potential determined. The *in vitro* antileishmanial activity was assessed against stationary promastigotes of *L. amazonensis* and results are shown in Table 1 as the compound concentration needed to inhibit 50% of *Leishmania* viability (IC_50_). Among the 21 evaluated compounds, nine were found to be biologically active. Compounds **2** (R = 2,3‐benzo), **3** (R = 3,4‐benzo), **13** (R = 4‐CH_3_), **17** (R = 3‐Cl), and **18** (R = 2‐Cl) displayed the best antileishmanial potential, with IC_50_ values below 15.0 μm. Interestingly, active compounds exhibit the highest calculated partition coefficient (ClogP) of the series, as shown in Table 1. ClogP is a well‐established measure of lipophilicity and the higher ClogP the greater the lipophilicity of a compound. Probably, the higher hydrophobic character of these compounds contributes to a more effective passage across parasite membranes and to a higher concentration at the site of action or even a better interaction with their target.^12^

**Table 1:** Antileishmanial activity, cytotoxicity parameters, and hydrophobic character of compounds **1**–**21**

Compound	IC_50_ a (μg/ml) ± *SD*	IC_50_ (μm) ± *SD*	CC_50_ b (μg/ml) ± *SD*	CC_50_ (μm) ± *SD*	SI^c^	ClogP^d^
**1**	12.5 ± 7.1	51 ± 29	36 ± 1	149 ± 4	3.0	3.07
**2**	5.1 ± 1.3	14.9 ± 3.9	12 ± 1	36 ± 4	3.0	5.53
**3**	5.1 ± 2.6	14.7 ± 7.5	24 ± 4	70 ± 12	5.0	5.53
**4**	> 50.0	ND^f^	ND	ND	ND	2.54
**5**	> 50.0	ND	ND	ND	ND	1.70
**6**	> 50.0	ND	ND	ND	ND	2.54
**7**	> 50.0	ND	ND	ND	ND	2.86
**8**	> 50.0	ND	ND	ND	ND	3.14
**9**	> 50.0	ND	ND	ND	ND	2.26
**10**	> 50.0	ND	ND	ND	ND	3.00
**11**	> 50.0	ND	ND	ND	ND	2.66
**12**	> 50.0	ND	ND	ND	ND	2.71
**13**	4.1 ± 2.6	14.9 ± 9.6	26 ± 4	96 ± 15	6.4	3.99
**14**	4.5 ± 0.3	16.6 ± 1.0	28 ± 8	102 ± 29	6.1	3.99
**15**	6.6 ± 2.0	24.1 ± 7.4	25 ± 7	92 ± 27	3.8	3.99
**16**	8.8 ± 0.9	28.1 ± 2.9	30 ± 1	97 ± 4	3.4	4.44
**17**	2.1 ± 0.5	6.7 ± 1.7	25 ± 1	80 ± 4	12.0	4.72
**18**	3.0 ± 0.4	9.5 ± 1.4	30 ± 4	97 ± 12	10.0	4.16
**19**	> 50	ND	ND	ND	ND	3.34
**20**	> 50	ND	ND	ND	ND	2.97
**21**	> 50	ND	ND	ND	ND	2.88
**AmpB** e	0.2 ± 0.0	0.2 ± 0.0	1 ± 0	1 ± 0	4.9	–

a IC_50_: 50% inhibitory concentration on *Leishmania amazonensis* promastigotes.

b CC_50_: 50% cytotoxicity concentration on murine macrophages.

c SI: selective index (CC_50_/IC_50_).

d ClogP = calculated partition coefficient, calculated using ACD/ChemSketch software (acdlabs.com).

e AmpB = Amphotericin B, positive control.

f ND = not determined.

The toxicity profile of active compounds was assessed on macrophages derived from BALB/c female mice and results are expressed in Table 1 as the compound concentration needed to inhibit 50% of macrophages viability (CC_50_). The selective toxicity is presented in Table 1 as selectivity index (SI), which is the ratio between CC_50_ and IC_50_. In the results, the two compounds with IC_50_ < 10.0 μm [**17**(R = 3‐Cl) and **18** (R = 4‐Cl)] presented the higher SI values, with results of 12.0 and 10.0, respectively. A high SI value is important to ensure the safety of mammalian cells, and SI values > 10.0 indicate a good selective toxicity.^13^ Other active compounds presented moderated or low selectivity against murine macrophages.

Besides the evaluation of antileishmanial potential of synthesized compounds against *L. amazonensis* promastigotes, their efficacy in treating infected macrophages with *Leishmania* amastigotes is desirable to be analyzed, as this parasite stage is responsible for the development of the disease in the infected mammalian hosts.^14^ Thus, compounds that showed IC_50_ values below 15.0 μm (**2**,** 3**,** 13**,** 17** and **18)** were selected for the evaluation of their antileishmanial potential against intramacrophage *L. amazonensis* amastigotes. Thus, murine macrophages previously infected with *L. amazonensis* were treated with different concentrations of selected compounds and their effect on reducing the percentage of infected macrophages, and the number of internalized parasites was evaluated (Table 2).

**Table 2:** Effect of selected compounds on the percentage of infected macrophages and on the number of amastigotes per macrophage

Compound	Concentration (μg/ml)	Percentage of infected macrophages after treatment	Reduction in percentage of infected macrophages (%)	Number of amastigotes per macrophage	Reduction in internalized parasites (%)
**2**	10	17.27	77	0.55	93
	5	31.45	58	2.27	70
	2.5	57.27	23	4.78	36
	0	74.00	–	7.50	–
**3**	15	20.90	71	0.75	88
	10	26.83	62	1.30	80
	5	38.20	46	2.40	63
	0	71.00	–	6.50	–
**13**	10	25.10	66	1.48	77
	5	26.62	60	1.72	74
	2.5	37.00	50	2.60	60
	0	74.00	–	6.50	–
**17**	15	8.85	88	0.34	95
	10	23.78	68	1.05	84
	5	38.00	49	2.60	60
	0	74.00	–	6.50	–
**18**	15	25.00	66	1.36	79
	10	43.75	41	2.90	55
	5	50.80	31	3.63	44
	0	74.00	–	6.50	–

The results of treatment of *L. amazonensis*‐infected macrophages indicated a dose‐dependent response, as the parasite burden and the number of infected macrophages decreased as the concentrations of selected compounds increased. All evaluated compounds were able to reduce the percentage of amastigotes per macrophage by more than 50% at concentrations above 5 μg/ml. Compound **13** (R = 4‐CH_3_) was the only one able to reduce by this percentage at a concentration below 5 μg/ml. This result indicates that the antileishmanial effect of evaluated compounds is different when one compares the results obtained using *in vitro* promastigotes and *in vitro* intramacrophage amastigotes. For instance, compound **13** reduced amastigotes viability by 50% at a lower concentration than compounds **17** and **18**, which present lower IC_50_ values against promastigote forms. In the same way, compound **13** was the only evaluated compound, which was able to reduce the percentage of infected macrophage by 50% at 2.5 μg/ml. On the other hand, only two compounds reduced the parasite burden by more than 90% at the evaluated concentrations: compound **2** (R = 2,3‐benzo), 93% at 10 μg/ml, and compound **17** (R = 3‐Cl), 95% at 15 μg/ml.

## 4 CONCLUSION

In this work, twenty‐one 1,3‐bis(aryloxy)propan‐2‐ols were synthesized and had their *in vitro* antileishmanial activity and cytotoxicity against mammalian macrophages evaluated. Several compounds exhibited effective antileishmanial profile against stationary promastigote forms of *L. amazonensis*, and an influence of compound lipophilicity on activity is suggested. Regarding the selective toxicity, most of the compounds displayed low selectivity against parasites as compared to macrophages, except compounds **17** and **18**, which presented good selective indexes (> 10.0). The treatment of infected macrophages showed that the most active compounds against *L. amazonensis* promastigotes displayed different profiles against amastigote forms. Altogether, these results indicate the potential of this class as a source of new antileishmanial agents and justify further studies, including on the mechanism of action of such compounds, which is presently unknown.

## CONFLICT OF INTEREST

The authors declared no conflict of interest.
