# Supplementary material for: 1,3‐Bis(aryloxy)propan‐2‐ols as potential antileishmanial agents
Source: Chem Biol Drug Des. 2017 Jun 28;90(5):981–6. doi: 10.1111/cbdd.13024 (PMC5697679; doi:10.1111/cbdd.13024)
Supplement: Supplementary file 1 [file CBDD-90-981-s001.docx]

**Supporting Information**

**1,3-Bis(aryloxy)propan-2-ols as potential antileishmanial agents**

Reactants were obtained from commercial suppliers and used without further purification. Melting points were determined on Microquímica MQAPF 301 apparatus. The ^1^H and ^13^C NMR spectra were obtained on a Bruker Avance DPX-200 spectrometer. The proton and carbon chemical shifts (δ) are given with respect to TMS. IR spectra were recorded on a Spectrum One, Perkin-Elmer ATR system. Column chromatography was performed on silica gel 60 0.063-0.200 mm/70-230 mesh Merck. Anhydrous DMSO was dried over 3Å molecular sieves.

***General procedure for synthesis of compounds 1-20***

To a stirred solution of NaOH (38 mmol) and the appropriate substituted phenol or naphthol (38 mmol) in water (30 mL) at 60ºC, epichloridrine (1 mL; 12.8 mmol) was added dropwise. The reaction mixture was stirred at 60ºC for 14 hours and then cooled to room temperature.

***1,3-Bisphenoxypropan-2-ol (1).***

The product was extracted with dichloromethane (3 x 30 mL) and washed with 1 M NaOH aqueous solution (2 x 20 mL) and water (5 x 50 mL). The organic phase was dried over anhydrous Na_2_SO_4_, filtered and concentrated to give a pale yellow solid with a yield of 77%. Mp: 77.8-79.0ºC (lit. 76-78ºC). IR *v* 3514, 3065, 3040, 2944, 2873, 1597, 1586, 1495, 1228, 1116, 1034, 749, 690 cm^-1^; ^1^H NMR (200 MHz, CDCl_3_) δ (ppm): 7.36-7.27 (m; 4H; Ar); 7.04-6.94 (m; 6H; Ar); 4.43 (asx; 1H; H-2; *J*=5.2 Hz); 4.21 (dd; 2H; H-1a; *J*=10.0 Hz; 4.4 Hz); 4.15 (dd; 2H; H-1b; *J*=10.0 Hz; 5.8 Hz); 2.74 (d; 1H; OH; *J*=5.2 Hz); ^13^C NMR (50 MHz, CDCl_3_) δ (ppm): 158.60 (Ar); 129.75 (Ar); 121.46 (Ar); 114.74 (Ar); 69.00 (C-2); 68.83 (C-1).

***1,3-bis(naphthalen-1-yloxy)propan-2-ol (2).***

The product was extracted with dichloromethane (3 x 30 mL) and washed with 1 M NaOH aqueous solution (2 x 20 mL) and water (5 x 50 mL). The organic phase was dried over anhydrous Na_2_SO_4_, filtered and concentrated to give a crude oil, which was purified by silica gel column chromatography eluting with Hexanes/EtOAc (9:1) to afford a pale brown solid with a yield of 54%. Mp: 63.4-64.7ºC (lit. 77-78ºC); IR *v* 3450, 3053, 2998, 2927, 2876, 1595, 1578, 1508, 1461, 1238, 1098, 1068 cm^-1^; ^1^H NMR (200 MHz, CDCl_3_) δ (ppm): 8.36-8.31 (m; 2H; Ar); 7.89-7.84 (m; 2H; Ar); 7.59-7.38 (m; 8H; Ar); 6.91 (d; 2H; Ar; *J*=7.4 Hz); 4.74 (aq; 1H; H-2; *J*=5.0 Hz); 4.46 (d; 4H; H-1; *J*=5.0 Hz); 2.73 (s; 1H; OH); ^13^C NMR (50 MHz, CDCl_3_) δ (ppm): 154.23 (Ar); 134.69 (Ar); 127.78 (Ar); 126.70 (Ar); 125.98 (Ar); 125.59 (Ar); 121.88 (Ar); 121.10 (Ar); 105.22 (Ar); 69.36 (C-1); 69.26 (C-2).

***1,3-Bis(naphthalen-2-yloxy)propan-2-ol (3).***

The product was extracted with dichloromethane (3 x 30 mL) and washed with 1 M NaOH aqueous solution (2 x 20 mL) and water (5 x 50 mL). The organic phase was dried over anhydrous Na_2_SO_4_, filtered and concentrated to give a crude solid, which was purified by silica gel column chromatography eluting with Hexanes/EtOAc (9:1) to afford a white solid with a yield of 65%. Mp: 112.4-114.5ºC (lit. 91-92ºC); IR *v* 3304, 3056, 2933, 1599, 1509, 1454, 1255, 1117, 1034 cm^-1^; ^1^H NMR (200 MHz, CDCl_3_) δ (ppm): 7.82-7.75 (m; 6H; Ar); 7.53-7.37 (m; 4H; Ar); 7.24 (s; 4H; Ar); 4.63-4.52 (m; 1H; H-2); 4.42-4.29 (m; 4H; H-1); 2.55 (s; 1H; OH); ^13^C NMR (50 MHz, CDCl_3_) δ (ppm): 156.64 (Ar); 134.70 (Ar); 129.79 (Ar); 129.48 (Ar); 127.88 (Ar); 127.04 (Ar); 126.70 (Ar); 124.11 (Ar); 118.83 (Ar); 107.43 (Ar); 69.23 (C-1); 69.09 (C-2).

***1,3-Bis(4-cyanophenoxy)propan-2-ol (4).***

A white solid was isolated by vacuum filtration with the yield of 92%. Mp: 163.9-165.6ºC. (lit. 120ºC). IR *v* 3501, 3070, 2936, 2219, 1605, 1574, 1506, 1450, 1253, 1173, 1003, 837 cm^-1^; ^1^H NMR (200 MHz, DMSO-*d_6_*) δ (ppm): 7.76 (d; 4H; Ar; *J*=8.4 Hz); 7.13 (d; 4H; Ar; *J*=8.4 Hz); 5.58 (s; 1H; OH); 4.17 (s; 5H; H-1 and H-2); ^13^C NMR (50 MHz, DMSO-*d_6_*) δ (ppm): 162.00 (Ar); 134.23 (Ar); 119.15 (CN); 115.66 (Ar); 102.97 (Ar); 69.54 (C-1); 67,07 (C-2).

***1,3-Bis(3-cyanophenoxy)propan-2-ol (5).***

A white solid was isolated by vacuum filtration with the yield of 63%. Mp: 92.5–93.6ºC; IR *v* 3503, 3090, 3044, 2927, 2234, 2225, 1607, 1576, 1477, 1252, 1129, 1142, 1027, 781, 677 cm^-1^; ^1^H NMR (200 MHz, CDCl_3_) δ (ppm): 7.45-7.18 (m; 8H; Ar); 4.45 (s; 1H; H-2); 4.19 (d; 4H; H-1; *J*=4.4 Hz); 2.76 (s; 1H; OH); ^13^C NMR (50 MHz, CDCl_3_) δ (ppm): 158.58 (Ar); 130.75 (Ar); 125.40 (Ar); 119.93 (Ar); 118.69 (CN); 117.75 (Ar); 113.51 (Ar); 69.07 (C-1); 68.56 (C-2).

***1,3-Bis(2-cyanophenoxy)propan-2-ol (6).***

A white solid was isolated by vacuum filtration with the yield of 63%. Mp: 141-141.8ºC; IR *v* 3499, 2957, 2228, 1597, 1487, 1448, 1287, 1104, 1023, 749 cm^-1^; ^1^H NMR (200 MHz, CDCl_3_) δ (ppm): 7.60-7.54 (m, 4H, Ar); 7.10-7.01 (m; 4H; Ar); 4.52-4.49 (m; 1H; H-2); 4.35 (d; 4H; H-1; *J*=5.0 Hz); 3.06 (s; 1H; OH); ^13^C NMR (50 MHz, CDCl_3_) δ (ppm): 160.12 (Ar); 134.86 (Ar); 133.83 (Ar); 121.72 (Ar); 116.56 (CN); 112.80 (Ar); 102.25 (Ar); 69.24 (C-1); 68.23 (C-2).

***1,3-Bis(4-nitrophenoxy)propan-2-ol (7).***

A yellow solid was isolated by vaccum filtration with the yield of 70%. Mp: 142.2-143.6ºC. (lit. 145ºC). IR *v* 3572, 3117, 2945, 1591, 1506, 1497, 1464, 1506, 1334, 1255, 1174, 1110, 847 cm^-1^; ^1^H NMR (200 MHz, Acetona-*d_6_*) δ (ppm): 8.21 (d; 4H; Ar; *J*=8.6 Hz); 7.19 (d; 4H; Ar; *J*=8.6 Hz); 4.90 (s; 1H; H-2); 4.44 (s; 1H; OH); 4.38 (s; 4H; H-1); ^13^C NMR (50 MHz, Acetona-*d_6_*): 165.04 (Ar); 142.54 (Ar); 126.66 (Ar); 115.85 (Ar); 70.97 (C-1); 68.88 (C-2).

***1,3-Bis(3-nitrophenoxy)propan-2-ol (8).***

A pale yellow solid was isolated by vacuum filtration with the yield of 89%. Mp: 113.7–114.5ºC. IR *v* 3341, 3095, 2926, 1616, 1582, 1532, 1483, 1518, 1341, 1243, 1125, 1141, 1025, 815, 734 cm^-1^; ^1^H NMR (200 MHz, Acetona-*d_6_*) δ (ppm): 7.72 (s; 4H; Ar); 7.61-7.53 (m; 2H; Ar); 7.45 (s; 2H; Ar); 4.84 (s; 1H; H-2); 4.42 (s; 1H; OH); 4.37 (s; 4H; H-1); ^13^C NMR (50 MHz, Acetona-*d_6_*) δ (ppm): 160.40 (Ar); 150.05 (Ar); 131.24 (Ar); 122.40 (Ar); 116.39 (Ar); 109.83 (Ar); 70.79 (C-1); 68.89 (C-2).

***1,3-Bis(2-nitrophenoxy)propan-2-ol (9).***

A green solid was isolated by vaccum filtration with the yield of 64%. Mp: 118.5-119ºC; IR *v* 3551, 3111, 2905, 1606, 1583, 1481, 1455, 1513, 1344, 1248, 1048, 1090, 1026, 736 cm^-1^; ^1^H NMR (200 MHz, Acetone-*d_6_*) δ (ppm): 7.84 (d; 2H; Ar; *J*=7.6 Hz); 7.66-7.62 (m; 2H; Ar); 7.42-7.38 (m; 2H; Ar); 7.15 (d; 2H; Ar; *J*=7.2 Hz); 4.73 (s; 1H; H-2); 4.42 (s; 5H; H-1 and OH); ^13^C NMR (50 MHz, Acetona-*d_6_*) δ (ppm): 152.76 (Ar); 141.07 (Ar); 135.22 (Ar); 126.05 (Ar); 121.59 (Ar); 115.97 (Ar); 71.17 (C-1); 68.72 (C-2).

***1,3-Bis(4-methoxyphenoxy)propan-2-ol (10).***

The product was extracted with dichloromethane (3 x 30 mL) and washed with 1 M NaOH aqueous solution (2 x 20 mL) and water (5 x 50 mL). The organic phase was dried over anhydrous Na_2_SO_4_, filtered and concentrated to give a white solid, with a yield of 89%. Mp: 91.1-92.7ºC. IR *v* 3492, 3105, 3069, 2954, 2939, 2880, 2837, 1505, 1455, 1220, 1116, 1032, 826 cm^-1^; ^1^H NMR (200 MHz, Acetona-*d_6_*) δ (ppm): 6.89-6.86 (m; 8H; Ar); 4.42 (d; 1H; OH; *J*=4.8 Hz); 4.30-4.20 (m; 1H; H-2); 4.11 (dd; 2H; H-1a; *J*=9.6 Hz; 4.0 Hz); 4.04 (dd; 2H; H-1b; *J*=9.6 Hz; 5.6 Hz); 3.73 (s; 6H; OCH_3_); ^13^C NMR (50 MHz, Acetona-*d_6_*) δ (ppm): 155.05 (Ar); 154.08 (Ar); 116.43 (Ar); 115.47 (Ar); 70.95 (C-1); 69.51 (C-2); 55.91 (OCH_3_).

***1,3-Bis(3-methoxyphenoxy)propan-2-ol (11).***

The product was extracted with dichloromethane (3 x 30 mL) and washed with 1 M NaOH aqueous solution (2 x 20 mL) and water (5 x 50 mL). The organic phase was dried over anhydrous Na_2_SO_4_, filtered and concentrated to give pure colorless oil, with a yield of 93%. IR *v* 3436, 3071, 2938, 2876, 2835, 1590, 1491, 1449, 1264, 1147, 1035, 759, 684 cm^-1^; ^1^H NMR (200 MHz, CDCl_3_) δ (ppm): 7.25-7.16 (m; 2H; Ar); 6.58-6.53 (m; 6H; Ar); 4.40 (asx; 1H; H-2; *J*=5.2 Hz); 4.17 (dd; 4H; H-1a; *J*=9.6 Hz; 4.6 Hz); 4.12 (dd; 4H; H-1b; *J*=9.6 Hz; 6.0 Hz); 3.80 (s; 6H; OCH_3_); 2.70 (d; 1H; OH; *J*=5.2 Hz); ^13^C NMR (50 MHz, CDCl_3_) δ (ppm): 161.04 (Ar); 159.84 (Ar); 130.18 (Ar); 107.09 (Ar); 106.83 (Ar); 101.28 (Ar); 68.94 (C-1 and C-2); 55.49 (OCH_3_).

***1,3-Bis(2-methoxyphenoxy)propan-2-ol (12).***

The product was extracted with dichloromethane (3 x 30 mL) and washed with 1 M NaOH aqueous solution (2 x 20 mL) and water (5 x 50 mL). The organic phase was dried over anhydrous Na_2_SO_4_, filtered and concentrated to give pure colorless oil, with a yield of 87%. IR *v* 3469, 3064, 2934, 2876, 2836, 1591, 1503, 1453, 1220, 1122, 1023, 738 cm^-1^; ^1^H NMR (200 MHz, CDCl_3_) δ (ppm): 7.02-6.90 (m; 8H; Ar); 4.45-4.40 (m; 1H; H-2); 4.24 (dd; 2H; H-1a; *J*=9.8 Hz; 4.8 Hz); 4.19 (dd; 2H; H-1b; *J*=9.8 Hz; 5.6 Hz); 3.86 (s; 6H; OCH_3_); 3.60 (d; 1H; OH; *J*=4.4 Hz); ^13^C NMR (50 MHz, CDCl_3_) δ (ppm): 150.00 (Ar); 148.41 (Ar); 122.23 (Ar); 121.17 (Ar); 115.19 (Ar); 112.15 (Ar); 71.09 (C-2); 68.29 (C-1); 56.00 (OCH_3_).

***1,3-Bis(4-methylphenoxy)propan-2-ol (13).***

The product was extracted with dichloromethane (3 x 30 mL) and washed with 1 M NaOH aqueous solution (2 x 20 mL) and water (5 x 50 mL). The organic phase was dried over anhydrous Na_2_SO_4_, filtered and concentrated to give a pale yellow solid, with a yield of 98%. Mp: 79.6-81.1ºC (lit. 89-89.5ºC); IR *v* 3514, 3068, 3027, 2983, 2947, 2923, 2860, 1609, 1585, 1509, 1230, 1118, 1013, 811 cm^-1^; ^1^H NMR (200 MHz, CDCl_3_) δ (ppm): 7.08 (d; 4H; Ar; *J*=8.2 Hz); 6.82 (d; 4H; Ar; *J*=8.2 Hz); 4.36 (aq; 1H; H-2; *J*=5.2 Hz); 4.13 (dd; 2H; H-1a; *J*=9.8 Hz; 4.6 Hz); 4.08 (dd; 2H; H-1b; *J*=9.8 Hz; 6.0 Hz); 2.67 (s; 1H; OH); 2.28 (s; 6H; CH_3_); ^13^C NMR (50 MHz, CDCl_3_) δ (ppm): 156.52 (Ar); 130.67 (Ar); 130.15 (Ar); 114.60 (Ar); 69.06 (C-1 and C-2); 20.67 (CH_3_).

***1,3-Bis(3-methylphenoxy)propan-2-ol (14).***

The product was extracted with dichloromethane (3 x 30 mL) and washed with 1 M NaOH aqueous solution (2 x 20 mL) and water (5 x 50 mL). The organic phase was dried over anhydrous Na_2_SO_4_, filtered and concentrated to give pure colorless oil, with a yield of 92%. IR *v* 3404, 3037, 2923, 2874, 1601, 1584, 1488, 1454, 1254, 1157, 1044, 767, 688 cm^-1^; ^1^H NMR (200 MHz, CDCl_3_) δ (ppm): 7.20 (t; 2H; Ar; *J*=7.8 Hz); 6.84-6.75 (m; 6H; Ar); 4.40 (asx; 1H; H-2; *J*=5.2 Hz); 4.16 (d; 2H; H-1a; *J*=4.6 Hz); 4.16 (d; 2H; H-1b; *J*=6.2 Hz); 2.73 (d; 1H; OH; *J*=5.0 Hz); 2.36 (s; 6H; CH_3_); ^13^C NMR (50 MHz, CDCl_3_): 158.62 (Ar); 139.81 (Ar); 129.46 (Ar); 122.26 (Ar); 115.59 (Ar); 111.63 (Ar); 69,01 (C-2); 68.80 (C-1); 21.70 (CH_3_).

***1,3-Bis(2-methylphenoxy)propan-2-ol (15).***

The product was extracted with dichloromethane (3 x 30 mL) and washed with 1 M NaOH aqueous solution (2 x 20 mL) and water (5 x 50 mL). The organic phase was dried over anhydrous Na_2_SO_4_, filtered and concentrated to give pure colorless oil, with a yield of 77%. IR *v* 3410, 3026, 2926, 2875, 1602, 1590, 1493, 1459, 1238, 1120, 1037, 745 cm^-1^; ^1^H NMR (200 MHz, Acetona-*d_6_*) δ (ppm): 7.14-7.10 (m; 4H; Ar); 6.98-6.80 (m; 4H; Ar); 4.48-4.36 (m; 2H; H-2 and OH); 4.24 (dd; 2H; H-1a; *J*=9.6 Hz; 4.6 Hz); 4.17 (dd; 2H; H-1a; *J*=9.6 Hz; 5.2 Hz); 2.20 (s; 6H; CH_3_); ^13^C NMR (50 MHz, Acetona-*d_6_*) δ (ppm): 158.00 (Ar); 131.39 (Ar); 127.82 (Ar); 127.34 (Ar); 121.34 (Ar); 112.09 (Ar); 70.33 (C-2); 69.55 (C-1); 16.42 (CH_3_).

***1,3-Bis(4-chlorophenoxy)propan-2-ol (16).***

The product was extracted with dichloromethane (3 x 30 mL) and washed with 1 M NaOH aqueous solution (2 x 20 mL) and water (5 x 50 mL). The organic phase was dried over anhydrous Na_2_SO_4_, filtered and concentrated to give a crude oil, which was purified by silica gel column chromatography eluting with Hexanes/EtOAc (9:1) to afford a white solid with a yield of 45%. Mp: 85.9-87.2ºC (lit. 87-89ºC); IR *v* 3525, 3068, 2928, 2872, 1594, 1580, 1488, 1448, 1232, 1120, 1008, 817 cm^-1^; ^1^H NMR (200 MHz, CDCl_3_) δ (ppm): 7.24 (d; 4H; Ar; *J*=9.0 Hz); 6.86 (d; 4H; Ar; *J*=9.0 Hz); 4.41-4.31 (m; 1H; H-2); 4.11 (d; 4H; H-1; *J*=4.6 Hz); 2.61 (d; 1H; OH; *J*=5.0 Hz); ^13^C NMR (50 MHz, CDCl_3_) δ (ppm): 157.18 (Ar); 126.66 (Ar); 126.49 (Ar); 116.04 (Ar); 69.18 (C-1); 68.86 (C-2).

***1,3-Bis(3-chlorophenoxy)propan-2-ol (17).***

The product was extracted with dichloromethane (3 x 30 mL) and washed with 1 M NaOH aqueous solution (2 x 20 mL) and water (5 x 50 mL). The organic phase was dried over anhydrous Na_2_SO_4_, filtered and concentrated to give a crude oil, which was purified by silica gel column chromatography eluting with Hexanes/EtOAc (9:1) to afford a colorless oil with a yield of 40%. IR *v* 3399, 3070, 2937, 2879, 1593, 1579, 1477, 1228, 1093, 1040, 764, 678 cm^-1^; ^1^H NMR (200 MHz, CDCl_3_) δ (ppm): 7.20 (t; 2H; Ar; *J*=8.0 Hz); 6.97-6.93 (m; 4H; Ar); 6.83-6.79 (m; 2H; Ar); 4.37 (s; 1H; H-2); 4.12 (s; 4H; H-1); 2.62 (s; 1H; OH); ^13^C NMR (50 MHz, CDCl_3_) δ (ppm): 159.22 (Ar); 135.15 (Ar); 130.53 (Ar); 115.21 (Ar); 113.19 (Ar); 114.74 (Ar); 69.00 (C-1); 68.73 (C-2).

***1,3-Bis(2-chlorophenoxy)propan-2-ol (18).***

The product was extracted with dichloromethane (3 x 30 mL) and washed with 1 M NaOH aqueous solution (2 x 20 mL) and water (5 x 50 mL). The organic phase was dried over anhydrous Na_2_SO_4_, filtered and concentrated to give a crude oil, which was purified by silica gel column chromatography eluting with Hexanes/EtOAc (9:1) to afford a white solid with a yield of 45%. Mp: 47.1-48.4ºC. IR *v* 3407, 3068, 2942, 2879, 1589, 1484, 1446, 1245, 1132, 1038, 743 cm^-1^; ^1^H NMR (200 MHz, CDCl_3_) δ (ppm): 7.36-7.17 (m; 4H; Ar); 6.99-6.91 (m; 4H; Ar); 4.46 (s; 1H; H-2); 4.26 (s; 4H; H-1); 2.90 (s; 1H; OH); ^13^C NMR (50 Mhz, CDCl_3_) δ (ppm): 154.07 (Ar); 130.49 (Ar); 128.05 (Ar); 123.26 (Ar); 122.26 (Ar); 114.06 (Ar); 69.78 (C-1); 68.69 (C-2).

***1,3-Bis(4-methoxycarbonylphenoxy)propan-2-ol (19).***

A white solid was isolated by vacuum filtration with the yield of 58%. Mp: 106.7-108ºC. IR *v* 3486, 3081, 2997, 2951, 2877, 1709, 1696, 1603, 1509, 1452, 1244, 1155, 1104, 845 cm^-1^; ^1^H NMR (200 MHz, Acetona-*d_6_*) δ (ppm): 7.96 (d; 4H; Ar; *J*=8.8 Hz); 7.07 (d; 4H; Ar; *J*=8.8 Hz); 4.72 (s; 1H; H-2); 4.39-4.28 (m; 5H; H-1 and OH); 3.84 (s; 6H; COOCH_3_); ^13^C NMR (50 MHz, Acetona-*d_6_*) δ (ppm): 166.96 (COOCH_3_); 163.73 (Ar); 132.28 (Ar); 123.71 (Ar); 115.30 (Ar); 70.44 (C-1); 69.08 (C-2); 52.10 (COOCH_3_).

***1,3-Bis(3-methoxycarbonylphenoxy)propan-2-ol (20).***

The product was extracted with dichloromethane (3 x 30 mL) and washed with 1 M NaOH aqueous solution (2 x 20 mL) and water (5 x 50 mL). The organic phase was dried over anhydrous Na_2_SO_4_, filtered and concentrated to give pure colorless oil with a yield of 40%. IR *v* 3485, 3077, 2951, 1716, 1585, 1488, 1274, 1099, 796, 731 cm^-1^; ^1^H NMR (200 MHz, Acetona-*d_6_*) δ (ppm): 7.48-7.44 (m; 4H; Ar); 7.33-7.25 (m; 2H; Ar); 7.14-7.09 (m; 2H; Ar); 4.58 (d; 1H; OH; *J*=5.0 Hz); 4.27-4.04 (m; 5H; H-1 and H-2); 3.74 (s; 6H; COOCH_3_); ^13^C NMR (50 MHz, Acetona-*d_6_*) δ (ppm): 167.10 (COOCH_3_); 160.00 (Ar); 132.53 (Ar); 130.62 (Ar); 122.68 (Ar); 120.46 (Ar); 115.87 (Ar); 70.43 (C-1); 69.20 (C-2); 51.25 (COOCH_3_).

***Procedure for synthesis of*** ***1,3-Bis(2-methoxycarbonylphenoxy)propan-2-ol (21).***

Sodium 2-methoxycarbonylphenolate was prepared by adding an aqueous NaOH solution (5 mL; 6.6 M) to a solution of methyl salycilate (5.0 g; 32.9 mmol) in acetone (10 mL). A white solid was isolated by vaccum filtration with the yield of 94%. To a stirred solution of sodium 2-methoxycarbonylphenolate (3.33 g; 19.1 mmol) in anhydrous DMSO (18 mL) at 60ºC, epichloridrine (0.5 mL; 6.4 mmol) was added dropwise. The reaction mixture was stirred at 60ºC for 48 hours. The solvent was evaporated with warm air and the residue was reconstituted in water. The product was extracted with toluene (3 x 30 mL) and washed with water (4 x 50 mL). The organic phase was dried over anhydrous Na_2_SO_4_, filtered and concentrated to give a crude oil, which was purified by silica gel column chromatography eluting with Hexanes/EtOAc (85:15) to afford pure colorless oil with a yield of 47%. IR *v* 3443, 2951, 1706, 1600, 1582, 1490, 1448, 1242, 1082, 752 cm-1; ^1^H NMR (200 MHz, CDCl_3_) δ (ppm): 7.82 (d; 2H; Ar; *J*=7.4 Hz); 7.47 (t; 2H; Ar; *J*=7.4 Hz); 7.07-6.97 (m; 4H; Ar); 4.56 (s; 1H; H-2); 4.38-4.35 (m; 4H; H-1); 3.88 (s; 7H; COOCH_3_ and OH); ^13^C NMR (50 MHz, CDCl_3_) δ (ppm): 166.78 (COOCH_3_); 158.90 (Ar); 134.05 (Ar); 131.91 (Ar); 121.14 (Ar); 120.34 (Ar); 114.76 (Ar); 70.36 (C-1); 68.41 (C-2); 52.25 (COOCH_3_).
